# Alterations of the gut microbiome are associated with epigenetic age acceleration and physical fitness

**DOI:** 10.1111/acel.14101

**Published:** 2024-02-27

**Authors:** Ferenc Torma, Csaba Kerepesi, Mátyás Jókai, Gergely Babszki, Erika Koltai, Balázs Ligeti, Regina Kalcsevszki, Kristen M. McGreevy, Steve Horvath, Zsolt Radák

**Affiliations:** ^1^ Research Institute of Sport Science Hungarian University of Sport Science Budapest Hungary; ^2^ Sports Neuroscience Division, Advanced Research Initiative for Human High Performance (ARIHHP), Faculty of Health and Sport Sciences University of Tsukuba Tsukuba Ibaraki Japan; ^3^ Laboratory of Exercise Biochemistry and Neuroendocrinology, Faculty of Health and Sport Sciences University of Tsukuba Tsukuba Ibaraki Japan; ^4^ Institute for Computer Science and Control (SZTAKI) Hungarian Research Network (HUN‐REN) Budapest Hungary; ^5^ Faculty of Information Technology and Bionics Pázmány Péter Catholic University Budapest Hungary; ^6^ Department of Biostatistics, Fielding School of Public Health University of California Los Angeles Los Angeles California USA; ^7^ Altos Labs, Cambridge Institute of Science Cambridge UK; ^8^ Waseda University Tokorozawa Japan

**Keywords:** aging clock, epigenetic clock, epigenetics, fitness, metagenomics

## Abstract

Epigenetic clocks can measure aging and predict the incidence of diseases and mortality. Higher levels of physical fitness are associated with a slower aging process and a healthier lifespan. Microbiome alterations occur in various diseases and during the aging process, yet their relation to epigenetic clocks is not explored. To fill this gap, we collected metagenomic (from stool), epigenetic (from blood), and exercise‐related data from physically active individuals and, by applying epigenetic clocks, we examined the relationship between gut flora, blood‐based epigenetic age acceleration, and physical fitness. We revealed that an increased entropy in the gut microbiome of physically active middle‐aged/old individuals is associated with accelerated epigenetic aging, decreased fitness, or impaired health status. We also observed that a slower epigenetic aging and higher fitness level can be linked to altered abundance of some bacterial species often linked to anti‐inflammatory effects. Overall our data suggest that alterations in the microbiome can be associated with epigenetic age acceleration and physical fitness.

## Graphical abstract

We show that aging is associated with an increase in the distance between nucleosomes, which can be used to predict a person's age and conduct age classification. We developed the first aging clock based on the nucleosome analysis of cell‐free DNA (cfDNA) extracted from blood plasma. Machine learning based on cfDNA distance distributions enabled the prediction of a person's age with the median absolute error of 3–3.5 years.

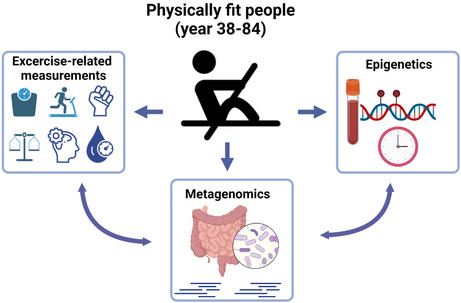

**Abbreviations**
BMI: body mass index
GripMax: maximal grip strength
HDL: high density lipoprotein
HMP: human microbiome project
JumpMax: maximal vertical jump
LDL: low density lipoprotein
SCFA: short‐chain fatty acids
VO2max: maximal relative oxygen uptake

## 1 INTRODUCTION

Currently, there are very limited options for increasing the maximum lifespan in humans. On the other hand, a substantial body of evidence, from several studies (Ortega et al., 2018; Radak et al., 2019), has consistently shown that a healthy lifestyle can increase the expected lifespan and reduce the incidence of lifestyle‐related diseases. DNA methylation‐based aging clocks (shortly epigenetic clocks) are developed to measure biological age that may be largely influenced by lifestyle among other environmental and genetic factors (Horvath & Raj, 2018; Levine et al., 2018; Quach et al., 2017). The first‐generation epigenetic clocks (such as Horvath's pan‐tissue clock, the blood‐based Hannum clock, and the Skin and Blood clock) can predict age accurately and exhibit associations with clinical biomarkers and mortality risk (Hannum et al., 2013; Horvath, 2013; Horvath et al., 2018). Second‐generation epigenetic clocks, such as PhenoAge, GrimAge, and DunedinPACE, show even stronger associations with mortality risk and some age‐related conditions (Belsky et al., 2022; Levine et al., 2018; Lu et al., 2019). Very recently, we developed DNAmFitAge which is based on genes that are related to physical fitness (McGreevy et al., 2023). It was shown that physically active people have a younger DNAmFitAge and experience better age‐related outcomes: lower mortality risk, coronary heart disease risk, and increased disease‐free status. DNA methylation‐based aging clocks are plastic and readily respond to lifestyle modifications and stress (Kankaanpää et al., 2022; Poganik et al., 2023). Another very plastic system in the human body is the microbiome, which is characterized by rapid change in childhood, followed by relatively long lifestyle‐associated stability, and finally age‐associated modification (Wilmanski et al., 2021). The results of a recent study using the microbiome profile of 9000 subjects revealed that with aging the microbiome flora is getting more and more unique, which is associated with immune regulation, inflammation, and longevity; moreover, over 85 years the high relative abundance of Bacteroides and having a low gut microbiome uniqueness measure were both associated with significantly decreased survival in the course of 4‐year follow‐up (Wilmanski et al., 2021). The microbiota of the gut is crucial for breaking down dietary nutrients, regulating intestinal and systemic immune responses, producing small molecules critical for intestinal metabolism, and generating several gasses that can modulate cellular function. Due to the complex function of the gut microbiome, the diversity of microbes can be defined as the range of different kinds of unicellular organisms, bacteria, archaea, protists, and fungi (Dunlap, 2001). However, the possible role or connection of the microbiome to epigenetic aging and physical fitness is still unknown. Recently attempts were made to create a pipeline to study the association of the architecture of microbiome and host diseases (Tierney et al., 2021). A metagenomics study revealed that the gut microbiome is a sex‐specific modulator of healthy aging in centenarians (Luan et al., 2024). Additionally, metagenomics aging clocks were developed based on taxonomic and functional profiling (Chen et al., 2022; Galkin et al., 2020; Gopu et al., 2024). Moreover, it appears that the well‐known difference in the mean lifespan of females and males could be associated with measurable differences in the microbiome and this sexual dimorphism in the microbiome (i.e., microgenderome) has high relevance to disease susceptibility (Ma & Li, 2019). Finally, it was proposed that alterations in the overall diversity of the gut microbiome had an epigenetic impact on the clock gene ARNTL which was involved in bipolar disorder (Bengesser et al., 2019). These approaches suggest the great potential of metagenomic investigations on human health, diseases, and aging. Here, we examine associations between microbiome flora, DNA methylation‐based aging, and the level of physical fitness.

## 2 METHODS

### 2.1 Inclusion and ethics

The study was approved by the National Public Health Center in accordance with the Helsinki Declaration and the regulations applicable in Hungary (25167–6/2019/EÜIG). The subjects of this study were volunteers who signed a written consent form to participate in the investigation.

### 2.2 Study population and physiology tests

Eighty volunteers participated in this study. Thirty‐eight of them were tested in the 2019 World Rowing Masters Regatta in Velence, Hungary. During the event, our research group had a tent with seven stages where we had completed the physiological tests, signed the acceptance of condition of participation, collected the samples, and fulfilled the documents of medical history. We did not have to reject any volunteer based on the received information of their medical history. The volunteers could come to our research area and take part in the sampling process. Participating in this study was on a voluntary basis, and the participants did not receive financial compensation. As compensation for their time, we sent the results of measurements to the volunteers via email. Additionally, 42 volunteers were tested at the Hungarian University of Sport Science, Budapest, Hungary with the same conditions as above described. Altogether, the sample was formed from 13 countries. Seventy people from Europe, three people from Australia, and seven people from North America. Our cohort was quite homogeneous regarding available lifestyle habits parameters. Among the 80 participants, only three smoked, four were vegetarian and one was on a gluten‐free diet.

Volunteers were tested, and based on the results of Chester's step, the maximal oxygen uptake was calculated and used as a measure of cardiovascular fitness. Maximum handgrip force is often used to measure the age‐associated decline in overall muscle strength. The dynamic strength of the legs was assessed by measuring the maximum vertical jump using a linear encoder. Body mass index was determined using the body composition monitor BF214 (Omron, Japan).

### 2.3 Measurement of irisin

Plasma irisin levels were quantified using commercially available ELISA kits (EK‐067–29, Irisin Recombinant, Phoenix Pharmaceuticals, Inc, Burlingame, USA). All samples from each subject were analyzed using the same plate (intra‐assay). The coefficients of variation for intra‐assay and inter‐assay were 4.1% and 15.2%, respectively.

### 2.4 Assessment of redox balance

Redox balance was calculated as the ratio of biological antioxidant power (BAP) to derivatives of reactive oxygen metabolites (d‐ROMs). BAP was measured by mixing ferric chloride with thiocyanate derivative in blood plasma samples. After incubation, the reduction of ferric ions was measured at 505 nm. BAP assays were performed using a FREE Carpe Diem analyzer. The total amount of organic hydroperoxides in blood was estimated using the d‐ROMs test, as described previously (Tsuchiya et al., 2008).

### 2.5 Microbiome assay

Stool samples were collected for analysis of gut microbiota. Participants were provided with instructions on proper methods for stool collection, and all necessary materials were included in a convenient specimen collection kit. The samples were stored at −80°C until further analysis. A frozen aliquot (200 mg) of each fecal sample was suspended in 250 mL of guanidine thiocyanate, 0.1 M Tris, pH 7.5, and 40 mL of 10% N‐lauroyl sarcosine. DNA extraction was then performed as previously described (Abraham et al., 2019), and the DNA concentration and molecular size were estimated using a nanodrop (Thermo Scientific) and agarose gel electrophoresis.

### 2.6 Illumina sequencing

Fecal DNA was used as input for the Illumina Nextera® XT DNA Sample Preparation Kit to construct indexed paired‐end DNA libraries, following the previously described method (Le Chatelier et al., 2013). DNA library preparation followed the manufacturer's instructions (Illumina). The workflow indicated by the provider was used for cluster generation, template hybridization, isothermal amplification, linearization, blocking and denaturing, and hybridization of the sequencing primers. The base‐calling pipeline (version IlluminaPipeline‐0.3) was used to process the raw fluorescent images and call sequences. One library (clone insert size 200 base pairs (bp)) was constructed for each of the first batch of 15 samples, two libraries with different clone insert sizes (135 and 400 bp) were constructed for each of the second batch of 70 samples, and one library (350 bp) was constructed for each of the third batch of 207 samples.

### 2.7 Bioinformatics analysis

The quality of raw and trimmed reads was assessed using FastQC and MultiQC. Low‐quality sequences were filtered and trimmed using Trimmomatic, discarding sequences with a minimum length of 36 and low‐quality base calls (phred score < 30). Reads aligning to the human reference genome (GRCh38) were removed to eliminate host contamination (using bowtie2 v2.4.2). Taxonomic characterization was performed using MetaPhlAn3 (Beghini et al., 2021), and pathway abundance and other molecular function profiles (such as GO) were estimated using the HUMAnN3 pipeline. We discarded a taxon if its average abundance over the 80 samples is less than 1%.

### 2.8 Measurement of DNA methylation

Epigenome‐wide DNA methylation was measured using the Infinium MethylationEPIC BeadChip (Illumina Inc., San Diego, CA) following the manufacturer's protocol. Briefly, 500 ng of genomic DNA was bisulfite‐converted using the EZ‐96 DNA Methylation MagPrep Kit (Zymo Research, Irvine, CA, USA) with the KingFisher Flex robot (Thermo Fisher Scientific, Breda, Netherlands). The samples were plated in a randomized order. Bisulfite conversion was performed according to the manufacturer's protocol with the following modifications: 15 μL MagBinding Beads were used for DNA binding, and the conversion reagent incubation was carried out in a cycle protocol of 16 cycles at 95°C for 30 s followed by 50°C for 1 h. After the cycle protocol, the DNA was incubated for 10 min at 4°C. Next, DNA samples were hybridized on the Infinium MethylationEPIC BeadChip (Illumina Inc., San Diego, CA) using 8 μL of bisulfite‐treated DNA as the starting material.

### 2.9 Quality control of the DNA methylation data

Quality control of the DNA methylation data was performed using the minfi, Meffil, and ewastools packages in R version 4.0.0. Samples that failed technical controls, including extension, hybridization, and bisulfite conversion according to Illumina's criteria, were excluded. Samples with a call rate <96% or with at least 4% of undetected probes were also excluded. Probes with a detection *p*‐value > 0.01 in at least 10% of the samples were considered undetected and excluded. Probes with a bead number < 3 in at least 10% of the samples were also excluded. The “noob” normalization method in R was used to quantify methylation levels.

### 2.10 DNA methylation (or epigenetic) aging clocks

Aging clocks were applied using Horvath's online age calculator (https://dnamage.genetics.ucla.edu/) and the DunedinPACE R package (https://github.com/danbelsky/DunedinPACE). We applied the Horvath pan‐tissue clock (Horvath, 2013), the blood‐based Hannum clock (Hannum et al., 2013), Skin and Blood clock (Horvath et al., 2018), PhenoAge clock (Levine et al., 2018), GrimAge clock (Lu et al., 2019), DNAmFitAge clock (McGreevy et al., 2023), and the DunedinPACE clock (Belsky et al., 2022). We calculated age acceleration as the residual, per sample, after fitting predicted ages to chronological ages (Thompson et al., 2018) (i.e., age acceleration is the deviation from the trend; Figure S2A–F). As expected, epigenetic clocks highly correlated with age and with each other in our cohort (Figure S2A–H) and age accelerations were independent of age (Figure S2I).

### 2.11 Statistical analysis

**FIGURE 1:**
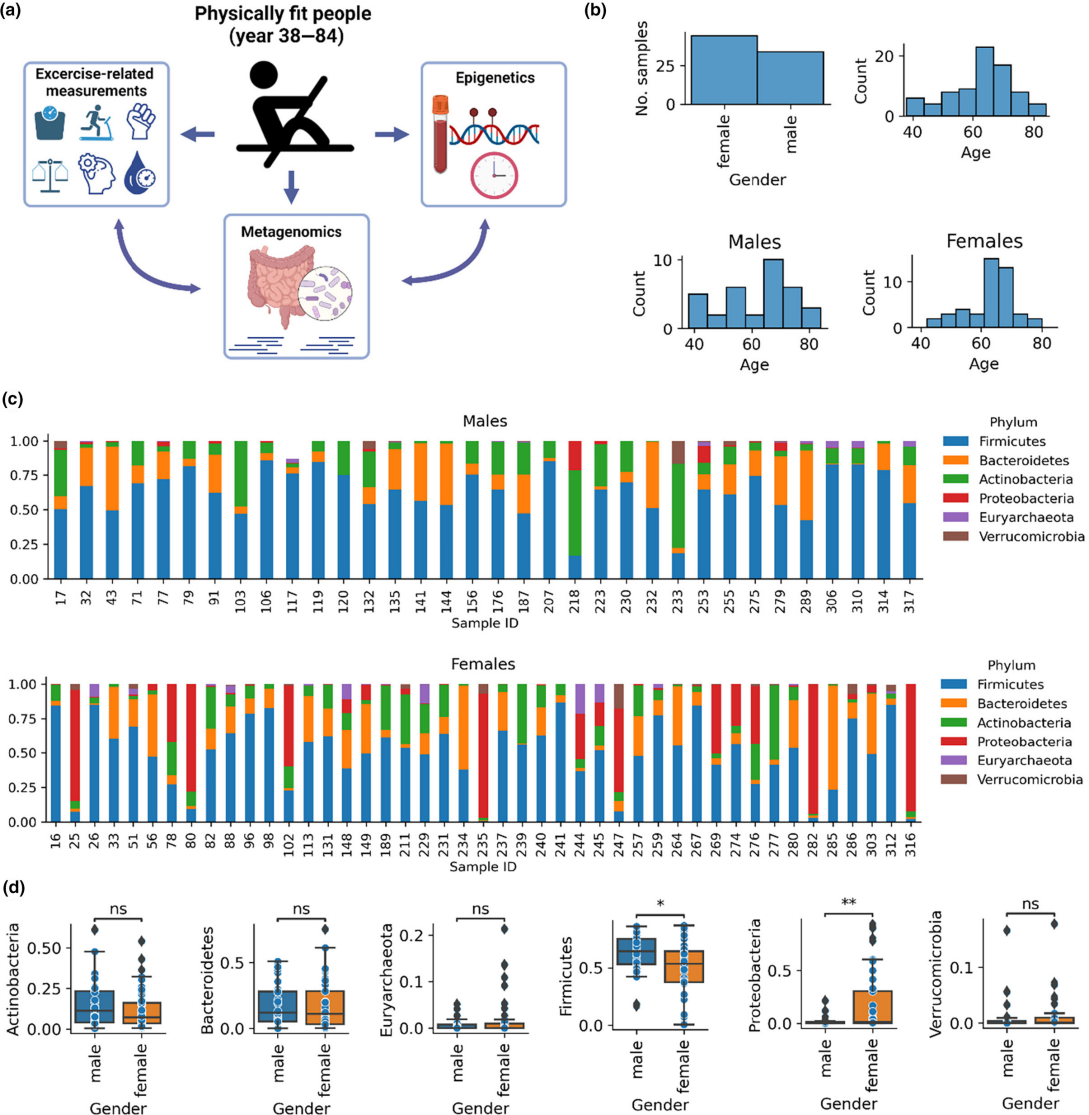
Characterization of the gut microbiome of middle‐aged/old physically active males and females. (a) Overview of the study. We collected metagenomics, epigenetics, and exercise‐related data from 80 physically active individuals with ages between 38 and 84 years and examined the relationship between epigenetic age acceleration, gut flora, and physical fitness. (b) Gender and age distribution of the study samples. 45 females and 34 males from 38 age groups were distributed between 38 and 84 years. (c) Phylum distributions for males and females, separately (only the abundant phyla are displayed). (d) Differences of mean relative abundances between males and females separated by phyla.

We used the Python packages for statistical analysis. Wilcoxon rank‐sum tests (two‐sided) were calculated for comparing two groups (Figure 1d) after we tested normality (D'Agostino and Pearson's test by using scipy.stats.normaltest) and homogeneity of variances (Levene's test by using scipy.stats.levene). Correlations were evaluated by Pearson correlation coefficient (*r*). If *p*‐values were indicated by an asterisk, we used the notations as follows: ns, *p* > 0.05; *, 0.01 < *p* ≤ 0.05; **, 0.001 < *p* ≤ 0.01; ***, *p* ≤ 0.001. We did not correct *p*‐values for multiple‐test comparison.

## 3 RESULTS

### 3.1 Characterization of the gut microbiome of physically active males and females

We collected metagenomic, epigenetic, and exercise‐related data from 80 physically active (i.e., non‐sedentary) people aged between 38 and 84 years (with 38 distinct age groups), and applying epigenetic clocks, we examined the relationship among the microbiome, epigenetic age acceleration, and physical fitness (Figure 1a). Characteristics of the study population are shown in Table S1. Females and older individuals are slightly overrepresented in our volunteer cohort (Figure 1b). First, we characterized the gut microbiome of physically active males and females in the phylum level. *Firmicutes* were the highest abundant taxa overall (mean: 0.556, std: 0.223) followed by *Bacteriodetes* (mean: 0.172, std: 0.223). We observed a remarkable difference in phylum and species distribution by gender (Figures 1c and S1, respectively). *Firmicutes* had a significantly lower relative abundance in females compared to males, while the relative abundance of *Proteobacteria* was significantly higher in females (Figure 1d). As males and females of our cohort expressed remarkable differences regarding the number of samples, microbiome composition, epigenetic age acceleration, and exercise‐related parameters, we conducted the data analysis separated by gender.

### 3.2 Associations of the microbiome and epigenetic aging clocks

**FIGURE 2:**
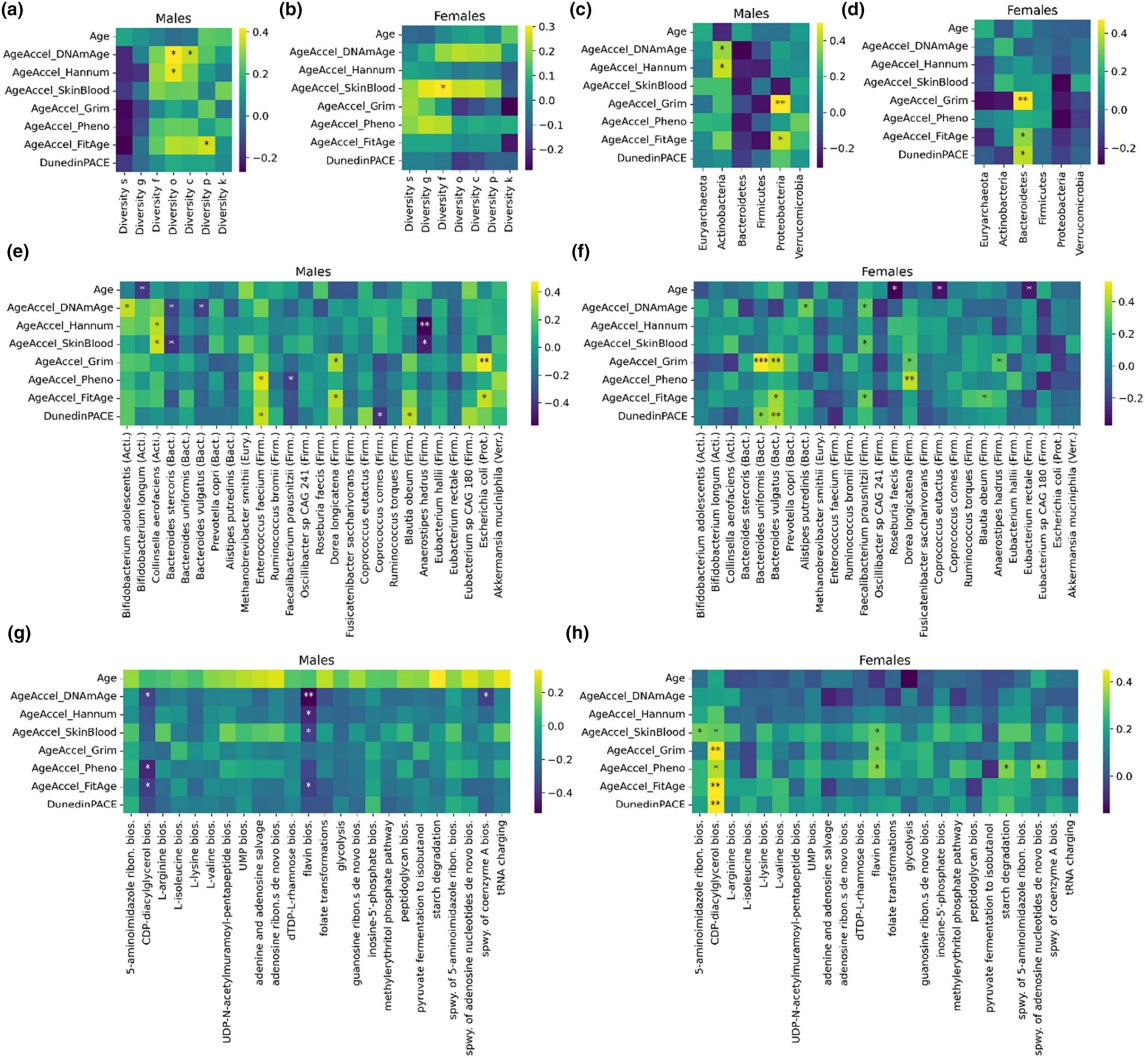
Associations of the gut microbiome and epigenetic aging clocks. (a, b) Correlations between the diversity of the microbiome (measured by Shannon entropy of the relative abundances) at the level of species (s), genus (g), family (f), order (o), class (c), phylum (p) and kingdom (k) and chronological age, age accelerations as well as the pace of aging. (c, d) Similar correlation analysis for the relative abundance of phyla. (e, f) Similar correlation analysis for the abundant species of the gut microbiome. Phyla are also displayed in parentheses abbreviated by the first four letters of the phylum. (g, h) Similar correlation analysis for the most abundant bacterial pathways in the gut microbiome (spwy., superpathway; bios., biosynthesis).

We investigated the relationships between epigenetic aging clocks and the results of shotgun sequencing of the microbiome (Figures 2 and S3). We calculated the diversity of the microbiome (measured by Shannon entropy of the relative abundances) at the seven taxonomic levels. We found significant positive correlations between age acceleration (i.e., the advanced biological age of an individual) and microbiome diversity for some epigenetic clocks at some taxonomic levels (four cases for males and one case for females; Figure 2a,b). In general, our data suggest that an increased entropy in the gut microbiome of physically active individuals may be associated with accelerated epigenetic aging.

For males, at the phylum level, the relative abundance of *Actinobacteria* and *Proteobacteria* both positively correlated with the age acceleration calculated by two‐two epigenetic clocks (Horvath's pan‐tissue DNAm age and the Hannum clock, as well as DNAmFitAge and GrimAge, respectively; Figure 2c). For females, at the phylum level, the relative abundance of *Bacteroidetes* positively correlated with the age acceleration by three epigenetic clocks (DNAmFitAge, GrimAge, and DunedinPACE; Figure 2d).

To refine the above results at the species level, we investigated the relationship between epigenetic clocks and the relative abundance of the abundant species. Instead of *p*‐value correction for multiple‐testing, we considered only strong associations (agreed by at least two epigenetic clocks and/or *p*‐value smaller than 0.01) in our conclusions. In males, the positive association of *Actinobacteria* and age acceleration seems to be driven by *Collinsella aerofaciens*, while the positive association of *Proteobacteria* can be linked to *Escherichia coli* (Figure 2e). Other remarkable positive associations with age acceleration or pace of aging were observed in the case of *Enterococcus faecium* and *Dorea longicatena*. Interestingly, strong negative associations with age acceleration also were found in the case of *Bacteroides stercoris* and *Anaerostipes hadrus*. In females, the positive association of *Bacteroidetes* phylum and age acceleration was clearly linked to two species, *Bacteroides uniformis* and *Bacteroides vulgatus* (Figure 2f). However, other remarkable positive associations with age acceleration were observed in the case of two *Firmicutes* species: *Faecalibacterium prausnitzii* and *Dorea longicatena*.

We also examined the relationship between bacterial pathways of the microbiome and epigenetic clocks. CDP‐diacylglycerol biosynthesis and flavin biosynthesis showed negative correlations with age acceleration and pace of aging in males (Figure 2g). Interestingly, the same pathways showed positive correlations with age acceleration and pace of aging in females (Figure 2h).

### 3.3 Associations of the microbiome and exercise‐related parameters

**FIGURE 3:**
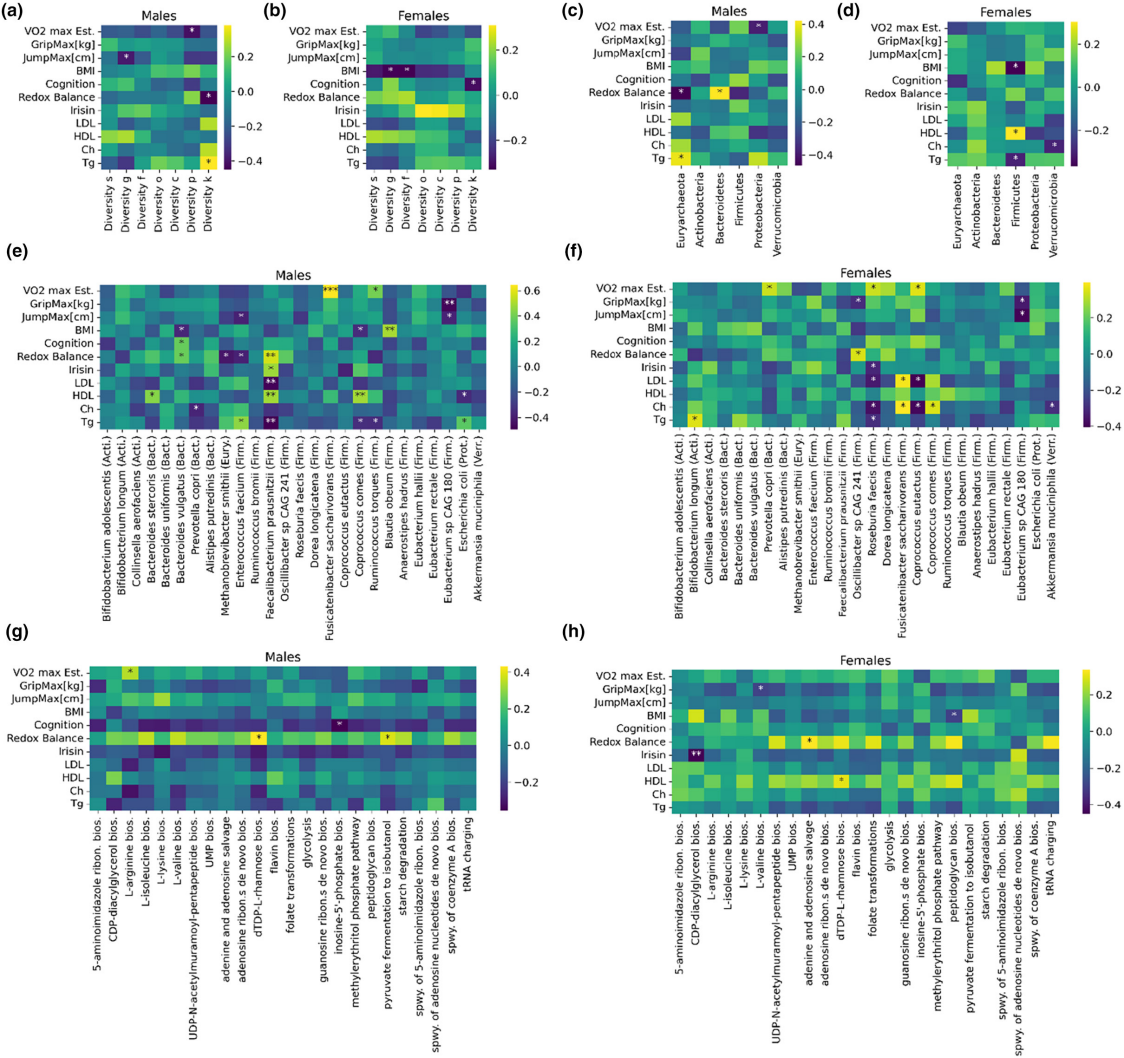
Associations of the microbiome and exercise‐related measurements. (a, b) Correlations between the diversity of the microbiome (measured by Shannon entropy of the relative abundances) at the level of species (s), genus (g), family (f), order (o), class (c), phylum (p) and kingdom (k) and exercise‐related parameters. (c, d) Similar correlation analysis for the relative abundance of phyla. (e, f) Similar correlation analysis for the abundant species of the gut microbiome. Phyla are also displayed in parentheses abbreviated by the first four letters of the phylum. (g, h) Similar correlation analysis for the most abundant bacterial pathways in the gut microbiome (spwy., superpathway; bios., biosynthesis).

We also investigated the relationship between exercise‐related measurements, relative abundance, and bacterial species‐catalyzed pathways (Figures 3 and S4). In males, we found significant negative correlations between microbial diversity and the parameters VO2max, JumpMax, and Redox Balance and positive correlations with triglyceride levels (Figure 3a). In females, we found significant negative correlations between microbial diversity and BMI and cognitive test performance (Figure 3b). Overall, our data suggest that an increased entropy in the gut microbiome of physically active middle‐aged/old individual is associated with accelerated epigenetic aging, decreased fitness, or impaired health status.

In males, at the phylum level, our statistical analysis revealed a positive relationship between *Euryarchaeota* and triglyceride levels, as well as, *Bacteroidetes* and redox balance, while a negative association was observed between *Euryarchaeota* and redox balance, as well as, *Proteobacteria* and VO2max (Figure 3c). In females, *Firmicutes* were negatively associated with BMI and triglyceride levels and positively associated with HDL levels (Figure 3d). Furthermore, the *Verrucomicrobiota* abundance was negatively associated with the cholesterol level.

We refined the above analysis at the species level and focused again on the highly significant associations (*p*‐value smaller than 0.01) or the cases when the consistency of multiple associations appeared. In males, the VO2max showed a remarkably high positive correlation with the abundance of *Fusicatenibacter saccharivorans* (Figure 3e). *Faecalibacterium prausnitzii* showed a high positive correlation with redox balance and HDL, and at the same time, remarkable negative correlations with LDL and triglyceride level. *Coprococcus comes* relative abundance expressed a positive association with HDL levels, while *Blautia obeum* showed a positive association with BMI. The genus *Eubacterium*, which consists of Gram‐positive bacteria, exhibited a negative relationship with GripMax and JumpMax.

In females, the abundance of *Roseburia faecis* showed negative associations with irisin, LDL, triglyceride, and cholesterol levels (Figure 3f). The genus *Eubacterium* exhibited negative associations with GripMax and JumpMax, similarly to males.

We examined the relationship between physical fitness‐related biomarkers and the molecular pathways that are catalyzed by bacterial species in the microbiome (Figure 2e). Redox balance showed a positive association with *dTDP‐L‐rhamnose biosynthesis* and *pyruvate fermentation to isobutanol* in males as well as with *adenine and adenosine salvage* pathway in females. Another remarkable observation is that, in females, irisin levels showed a low correlation to cdp‐diacylglycerol pathway.

### 3.4 Associations of diet and medication using with exercise‐related parameters, aging clocks, and the microbiome in physically active individuals

As we had questionnaires about diet and medication use, we examined the associations of diet and medication with the remaining measurements of the study. We required at least four cases to include a condition for a comparison. Accordingly, we examined exercise‐related parameters, aging clocks, and the microbiome for vegetarian (*n* = 5) versus normal diet (*n* = 74), asthma yes (*n* = 4) or no (*n* = 76), and blood pressure lowering medication yes (*n* = 12) or no (*n* = 68; Figure S5). We observed that a vegetarian diet was associated with higher levels of HDL compared to the normal diet. Age predictions of the epigenetic clocks were positively associated with the use of blood pressure lowering medications, with the strongest association presented by DNAmFitAge. In contrast, GripMax[Kg] is negatively associated with the use of blood pressure lowering medications. We also observed significant associations between the abundance of some taxa with asthma and blood pressure lowering medications. Most remarkably, the abundance of *Roseburia faecis* was significantly lower in individuals who used blood pressure lowering medications compared to the remaining ones (*p* < 0.01). Interestingly, the vegetarian diet, asthma, and blood pressure lowering medication use were not significantly associated with age acceleration determined by any aging clocks in our analysis. This may be due to the low sample sizes with the conditions.

## 4 DISCUSSION

The microbiome is highly plastic to acute nutritional and exercise interventions, or health status and relatively stable because longitudinal studies suggest that the composition of intestinal microbiota does not drastically change in adults within the periods examined (Bajzer & Seeley, 2006). Males and females have differences in lifespan, sex hormones, muscle mass, VO2max, and the power of the immune system (Lotter & Altfeld, 2019; Ma & Li, 2019); therefore, our observation on the difference between male and female microbiome could be expected. Early results of the Human Microbiome Project (HMP) revealed that the highest abundant phyla in the gut microbiome of the general healthy population are Bacteroidetes and Firmicutes (Huttenhower et al., 2012). Consistently, these two phyla were the most abundant in our physically active cohort; however, the proportion of the two phyla was the opposite in our cohort compared to the general healthy population of the HMP project. Another study reported the dominance of *Firmicutes* with the considerable abundance of *Bacteroidetes*, *Actinobacteria*, and *Proteobacteria* in the gut flora of individuals between the ages 40 and 80 years that is consistent with our results in approximately the same age group (Odamaki et al., 2016). Interestingly, in our cohort, several (mostly female) metagenomes contained high abundances of *Proteobacteria* (Figure 1c) due to the high abundance of *Escherichia coli* (Figure S1). *E. coli* was presented at >0.1% abundance in 20% of stool microbiomes of the physically active individuals (>0% abundance in 65% of them). The HMP presented a remarkable similarity with *E. coli* abundance >0.1% in 15% of stool microbiomes (>0% abundance in 61%). Altogether, the microbiome composition of our middle‐aged/old physically active cohort recapitulated some previous findings analyzed healthy individuals.

The composition of the gut flora changes with age even with considering only healthy subjects. For example, a machine learning model predicted the age of 20–70 years old healthy subjects with the MAE = 0.591 and *r* = 0.52. In contrast, here we observed only a marginal change with age among physically active people between ages 38 and 84 years (no changes in phylum composition, small change in species‐level composition). However, age‐related changes may show different rates during life. The analysis of Japanese subjects revealed only two transition points: the gut microbiome changed with age as it matured in subjects younger than 20 years and changed again in subjects older than 70 into the the elderly type (Odamaki et al., 2016). The analysis of very healthy individuals showed that the gut microbiome of healthy aged Chinese was similar to that of the healthy young (Bian et al., 2017). Our study involved physically active people implying a selection of mostly healthy and non‐obese subjects. That may explain why we observed only a small change in the composition of the gut microbiome of physically active subjects between the ages 39 and 84.

The *Proteobacteria* phylum contains a number of pathogens such as *Brucella*, *Rickettsia*, *Shigella*, *Salmonella*, *Yersinia*, *Helicobacter*, and *Escherichia*. Therefore, it was suggested that the increased prevalence of *Proteobacteria* is a potential diagnostic signature of dysbiosis and risk of disease (Shin et al., 2015). We found a positive correlation between *Proteobacteria* and age acceleration in males with a species‐level dominance of *Escherichia coli*. Therefore, it is possible that the infections of some pathogen *Escherichia coli* strain accelerate epigenetic aging. This would be consistent with previous findings where accelerated epigenetic aging was reported in the case of other pathogen infections such as HIV and SARS‐CoV‐2 (Cao et al., 2022; Horvath & Levine, 2015; Poganik et al., 2023).

*Collinsella aerofaciens* was identified as pro‐inflammatory bacteria in rheumatoid arthritis, psoriasis, Crohn's disease, inflammatory bowel disease, atherosclerosis, non‐alcoholic steatohepatitis, type 2 diabetes, and COVID‐19 (Kwon et al., 2023). Both of these species are associated with accelerated epigenetic aging in males according to our findings.

We observed a negative correlation between epigenetic age acceleration and the relative abundance of *Anaerostipes hadrus*, which has been identified as an anti‐inflammatory, butyrate‐producing species, and butyrate has been suggested to beneficially affect gut health (Kant et al., 2015).

We found remarkable positive associations with age acceleration for *Faecalibacterium prausnitzii* and *Dorea longicatena*. In contrast, the gut microbiota analysis of Crohn disease patients showed that *Faecalibacterium prausnitzii* was an anti‐inflammatory bacteria (Sokol et al., 2008); however, Dorea might have either pro or anti‐inflammatory roles in multiple sclerosis (Shahi et al., 2017).

In females, we also observed a remarkable positive correlation with age acceleration for the relative abundance of *Dorea longicatena*, which was linked to metabolic risk markers in obesity (Brahe et al., 2015), and its level was higher in overweight subjects (Companys et al., 2021). Epigenetic age acceleration was linked to obesity in previous studies (Levine et al., 2018; Quach et al., 2017). In females, we also observed a positive association between the *Bacteroidetes* phylum and age acceleration that was clearly linked to two species: *Bacteroides uniformis* and *Bacteroides vulgatus*. A gut microbiome analysis linked *Bacteroides vulgatus* with ulcerative colitis severity (Mills et al., 2022).

*Fusicatenibacter saccharivorans* highly correlated with VO2max in males, and this Gram‐positive bacteria reportedly reduced inflammation (Takeshita et al., 2016) and contributed to the production of short‐chain fatty acids (SCFA) (Xie et al., 2021), which are crucial to the regulation of tight junction proteins involved in the permeability of the epithelial barrier in the colon that are associated with obesity and insulin resistance (Cani et al., 2008). Through receptor activation, gut‐derived SCFA is an active player in signaling and metabolic organ‐to‐organ communication. However, it is not known why *Fusicatenibacter saccharivorans* level is positively linked to cardiovascular fitness. One possible mechanism could be that *Fusicatenibacter* produces butyrate which supplementation can activate peroxisome proliferator‐activated receptor‐gamma coactivator‐1 alpha levels and the activities of AMP kinase and p38 in the skeletal muscle of mice (Gao et al., 2009). Higher mitochondrial content can readily lead to increased VO2max; however, it is not known whether a similar mechanism could happen in humans.

The genus *Eubacterium* sp. *CAG 180* exhibited a negative relationship with GripMax and JumpMax in both genders, but there are no associations for other abundant *Eubacteria* species (*Eubacterium rectale* and *Eubacterium hallii*). While the greater abundance of some *Eubacteria* species such as *Eubacterium ventriosum* suggested having health benefits (Amamoto et al., 2022), there is no report for *Eubacterium* sp. *CAG 180*.

Redox balance showed a positive association with *dTDP‐L‐rhamnose biosynthesis* and *pyruvate fermentation to isobutanol* in males as well as with *adenine and adenosine salvage* pathway in females. This observation suggests that redox balance in a cell has a positive effect on the abovementioned molecular pathways, potentially indicating a regulatory role of redox balance in various cellular and metabolic pathways. VO2max was correlated with L‐arginine biosynthesis in males, which fits well with the result of the study which reported increased VO2max after L‐arginine supplementation (Rezaei et al., 2021).

It was also observed that irisin levels were negatively related to cdp‐diacylglycerol pathway in females. The cdp‐diacylglycerol pathway is involved in the synthesis of phosphatidylcholine, a major component of cell membranes. A negative correlation between irisin levels and this pathway suggests that higher levels of irisin may be associated with lower activity or expression of genes involved in the cdp‐diacylglycerol pathway. These correlations indicate potential connections between cognitive performance, hormone levels, and specific metabolic pathways.

## 5 LIMITATIONS AND STRENGTHS

Besides the novelty our investigation also has limitations. One of the limitations is the plasticity of DNA methylation‐based aging clocks and gut microbiomes as well. Both systems are strongly influenced by long‐term and short‐term lifestyle factors; hence, the presented results reflect the relationships at the time point of sampling. We investigated only middle‐aged/old physically active individuals without a young or sedentary group. Therefore, our conclusions should be restricted mainly to middle‐aged/old, physically active individuals. However, despite this limitation, the present study could be one of the first that investigates the relationship between DNA methylation‐based aging clocks and gut microbiome, revealing that for physically active individuals slower epigenetic aging and higher physical fitness levels can be linked to altered abundance of some bacterial species often linked to anti‐inflammatory effects.

## 6 CONCLUSIONS

In conclusion, our data not only confirmed that gut microbiome‐located bacterial species are modulating a wide range of physiological and biochemical processes, but our results revealed that there are associations between the microbiome and DNA methylation‐based aging clocks. Overall our data suggest that alterations in the microbiome can be associated with epigenetic age acceleration and physical fitness. These findings could be important new mosaics of the beneficial effects of exercise on health and well‐being.

## AUTHOR CONTRIBUTIONS

Conceptualization: Z.R. Methodology: F.T. C.K. S.H. K.M, and Z.R. Investigation: M.J., F.T., E.K., G.B., P.B., and Z.R. Formal analysis: K.M., F.T., R.K. C.K., and S.H. Writing—original draft: Z.R. and C.K. Writing—review and editing: C.K., S.H., B.S., and Z.R. Supervision: Z.R.

## CONFLICT OF INTEREST STATEMENT

None declared.

## Supporting information


Figure S1.
Figure S2.
Figure S3.
Figure S4.
Figure S5.
Table S1.

Table S2.

## Data Availability

Sample characteristics, epigenetic clock results, physical fitness parameters, abundances of the phylum, and the species level are available in Table S2. All data and codes are available upon request.
