# Supplementary material for: Alterations of the gut microbiome are associated with epigenetic age acceleration and physical fitness
Source: Aging Cell. 2024 Feb 27;23(4):e14101. doi: 10.1111/acel.14101 (PMC11019127; doi:10.1111/acel.14101)
Supplement: Supplementary file 1 — Figure S1. Figure S2. Figure S3. Figure S4. Figure S5. Table S1. [file ACEL-23-e14101-s002.docx]

**SUPPLEMENTARY FIGURES**

**SUPPLEMENTARY TABLE S1.** Characteristics of the study population.

|  | **Middle-aged (38 - 64 years)** | | | | **Old (65 - 85 years)** | | | |
| --- | --- | --- | --- | --- | --- | --- | --- | --- |
|  | **Females** | | **Males** | | **Females** | | **Males** | |
|  | **N (Min,Max)** | **Mean (Std)** | **N (Min,Max)** | **Mean (Std)** | **N (Min,Max)** | **Mean (Std)** | **N (Min,Max)** | **Mean (Std)** |
| **Age** | 21 (42.0,63.0) | 56.4 (6.8) | 15 (38.0,59.0) | 50.1 (7.0) | 24 (65.0,80.0) | 68.7 (4.4) | 19 (65.0,84.0) | 71.3 (5.2) |
| **VO2 max Est.** | 21 (26.3,55.1) | 39.7 (8.0) | 15 (37.3,72.4) | 47.3 (8.7) | 24 (21.0,43.8) | 32.5 (6.8) | 19 (21.6,50.3) | 38.8 (8.3) |
| **GripMax[kg]** | 21 (20.7,47.9) | 33.1 (6.3) | 15 (40.2,65.2) | 54.0 (6.5) | 24 (15.1,38.3) | 27.0 (5.9) | 19 (22.5,60.3) | 42.5 (9.6) |
| **JumpMax[cm]** | 21 (15.7,33.3) | 26.4 (5.1) | 15 (23.3,48.1) | 35.9 (7.0) | 24 (13.5,33.7) | 21.5 (4.9) | 19 (10.3,43.4) | 26.5 (8.0) |
| **BMI** | 21 (19.5,29.6) | 24.2 (2.8) | 15 (21.6,29.9) | 25.2 (2.1) | 24 (19.0,31.4) | 24.8 (3.4) | 19 (16.1,33.8) | 25.7 (4.0) |
| **Cognition** | 21 (5.0,9.0) | 6.7 (1.2) | 15 (4.0,9.0) | 6.6 (1.4) | 24 (0.0,8.0) | 5.7 (1.5) | 19 (4.0,8.0) | 6.1 (1.3) |
| **Redox Balance** | 16 (4.3,7.1) | 5.3 (0.7) | 13 (4.6,8.1) | 6.2 (1.1) | 21 (3.7,7.5) | 5.3 (0.9) | 16 (4.3,8.5) | 6.0 (1.0) |
| **Irisin** | 19 (9.4,17.7) | 12.6 (2.3) | 9 (9.7,15.9) | 13.2 (2.0) | 21 (8.6,15.4) | 11.5 (1.7) | 14 (9.3,18.4) | 12.2 (2.4) |
| **LDL** | 21 (2.3,5.3) | 3.4 (0.7) | 15 (2.6,4.6) | 3.4 (0.7) | 24 (2.4,6.1) | 4.0 (0.9) | 19 (2.4,5.0) | 3.7 (0.8) |
| **HDL** | 21 (1.0,2.6) | 1.8 (0.4) | 15 (1.1,2.4) | 1.7 (0.4) | 24 (1.0,2.5) | 1.8 (0.4) | 19 (1.0,2.4) | 1.5 (0.4) |
| **Ch** | 21 (4.2,7.7) | 5.8 (1.0) | 15 (4.5,6.8) | 5.7 (0.8) | 24 (4.9,9.2) | 6.6 (1.2) | 19 (4.1,7.8) | 6.0 (1.0) |
| **Tg** | 21 (0.7,2.1) | 1.3 (0.4) | 15 (0.6,2.8) | 1.3 (0.6) | 24 (0.7,3.4) | 1.6 (0.9) | 19 (0.8,3.8) | 1.7 (0.8) |
| **DNAmAge** | 21 (42.4,64.5) | 53.8 (5.7) | 15 (31.3,60.9) | 48.0 (7.4) | 24 (50.3,76.1) | 62.1 (6.0) | 18 (59.3,78.6) | 66.3 (5.4) |
| **DNAmAgeHannum** | 21 (30.9,57.0) | 46.1 (7.7) | 15 (28.0,53.0) | 42.4 (6.1) | 24 (47.7,68.5) | 55.3 (5.2) | 18 (54.1,68.3) | 60.5 (3.5) |
| **DNAmAgeSkinBloodClock** | 21 (42.0,64.8) | 56.2 (6.7) | 15 (36.3,59.3) | 50.1 (6.9) | 24 (59.6,80.4) | 67.2 (4.8) | 18 (60.8,77.6) | 69.8 (4.7) |
| **DNAmGrimAge** | 21 (43.4,63.5) | 54.6 (5.2) | 15 (44.7,60.4) | 52.2 (5.3) | 24 (60.3,76.7) | 66.2 (5.0) | 18 (60.6,83.9) | 70.8 (5.7) |
| **DNAmPhenoAge** | 21 (32.6,59.9) | 44.4 (7.7) | 15 (28.7,49.5) | 39.5 (6.1) | 24 (43.1,74.3) | 55.0 (7.7) | 18 (47.1,73.3) | 59.4 (6.9) |
| **DNAmFitAge** | 21 (43.0,70.2) | 59.0 (6.9) | 15 (43.8,62.3) | 51.8 (5.1) | 24 (65.1,88.2) | 73.3 (5.9) | 18 (61.2,91.2) | 73.4 (7.3) |
| **DunedinPACE** | 21 (0.7,1.0) | 0.9 (0.1) | 15 (0.8,1.1) | 0.9 (0.1) | 24 (0.7,1.2) | 1.0 (0.1) | 19 (0.8,1.3) | 1.0 (0.1) |
| **AgeAccel_DNAmAge** | 21 (-8.4,6.7) | 0.4 (3.8) | 15 (-8.3,6.4) | -0.4 (4.9) | 24 (-10.5,9.5) | -0.9 (4.6) | 18 (-5.4,6.6) | 1.0 (3.3) |
| **AgeAccel_Hannum** | 21 (-8.1,5.6) | -0.5 (3.4) | 15 (-3.4,6.0) | 1.0 (3.2) | 24 (-8.4,3.4) | -1.3 (3.1) | 18 (-3.4,5.5) | 1.6 (2.8) |
| **AgeAccel_SkinBlood** | 21 (-5.9,4.6) | 0.1 (2.5) | 15 (-4.6,5.6) | -0.3 (3.4) | 24 (-6.1,5.0) | 0.1 (2.8) | 18 (-4.0,4.4) | 0.1 (2.4) |
| **AgeAccel_Grim** | 21 (-5.2,1.6) | -1.8 (2.1) | 15 (-3.9,10.7) | 1.1 (3.7) | 24 (-5.1,5.6) | -0.4 (2.8) | 18 (-2.9,11.0) | 1.7 (3.9) |
| **AgeAccel_Pheno** | 21 (-11.6,10.1) | -0.3 (5.5) | 15 (-3.1,6.7) | 0.5 (2.4) | 24 (-9.3,8.4) | -0.8 (5.4) | 18 (-12.6,9.2) | 1.0 (5.5) |
| **AgeAccel_FitAge** | 21 (-4.9,4.3) | -0.2 (2.3) | 15 (-7.7,5.4) | -0.9 (4.0) | 24 (-4.9,7.2) | 1.7 (3.4) | 18 (-9.3,6.5) | -1.2 (4.4) |

**
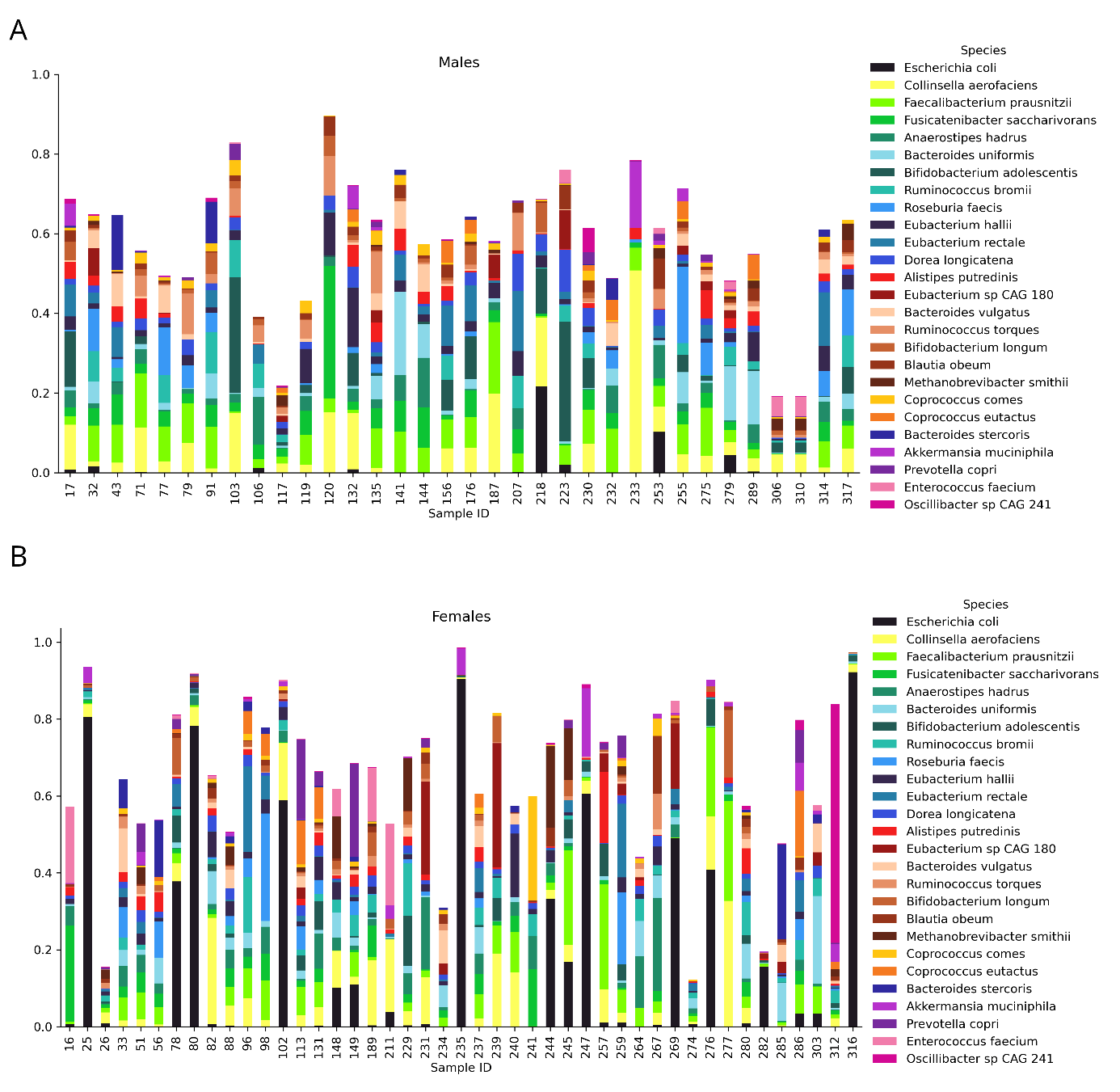
**

**SUPPLEMENTARY FIGURE S1** Species distributions of the gut microbiome of our cohort. (A) males (B) females. Only the abundant species are displayed. This figure is related to Fig. 1C.

**
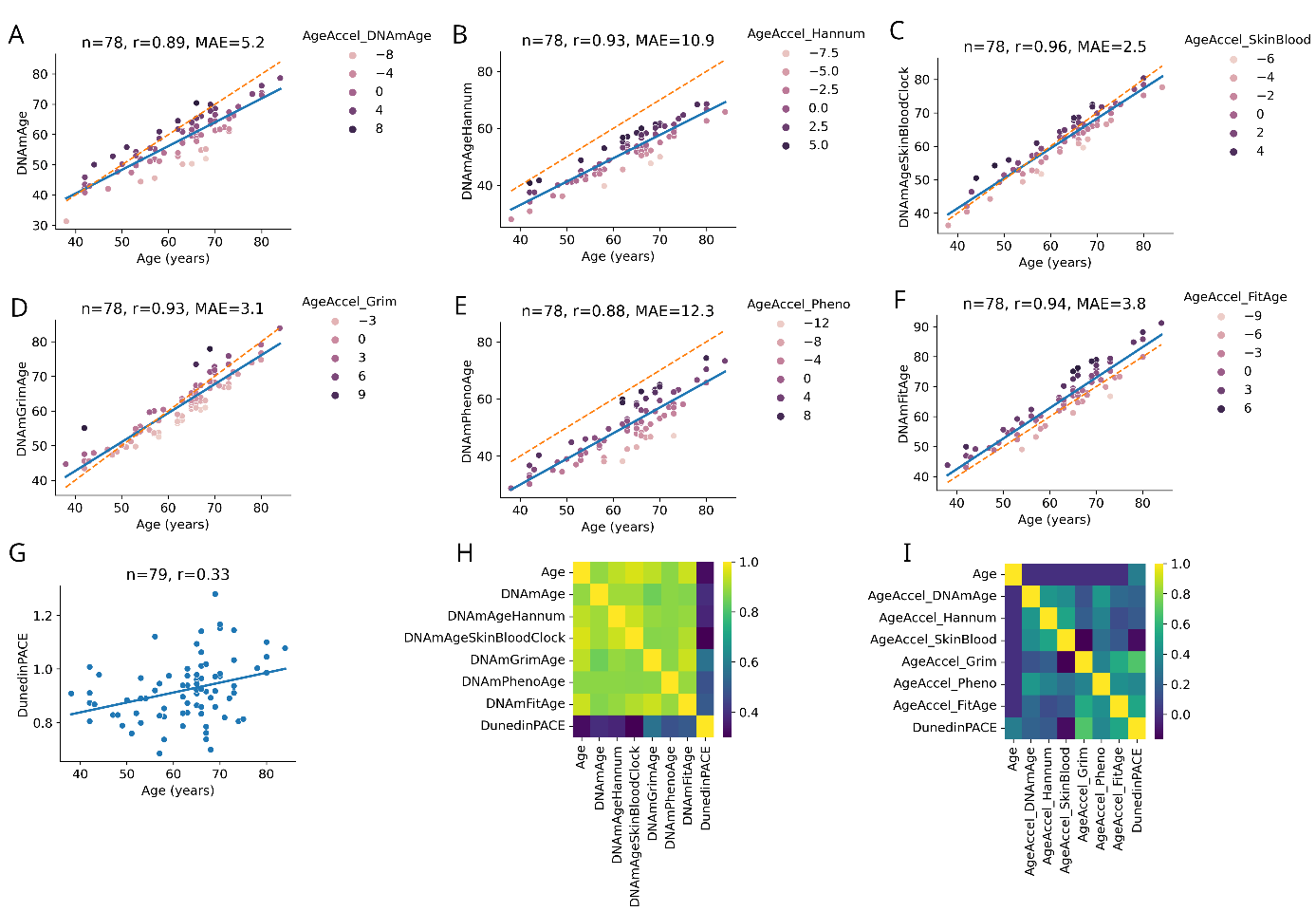
**

**SUPPLEMENTARY FIGURE S2** Application of epigenetic clocks in our cohort. (A-G) Predicted age (i.e. epigenetic age) of the six epigenetic clocks as well as the pace of aging by DunedinPACE. Number of samples (n), Pearson correlation coefficient (r) and mean absolute error (MAE) are indicated for each clock. Linear regression line (solid blue lines) of the predicted ages is also shown. Age acceleration (i.e. the deviation from the trend) is illustrated by coloring. The dashed orange line is the diameter (x=y). (H) Pearson correlations among age and the predicted values of the epigenetic clocks. (I) Pearson correlations among age, age accelerations and the pace of aging. The significance of correlations is not indicated.

**
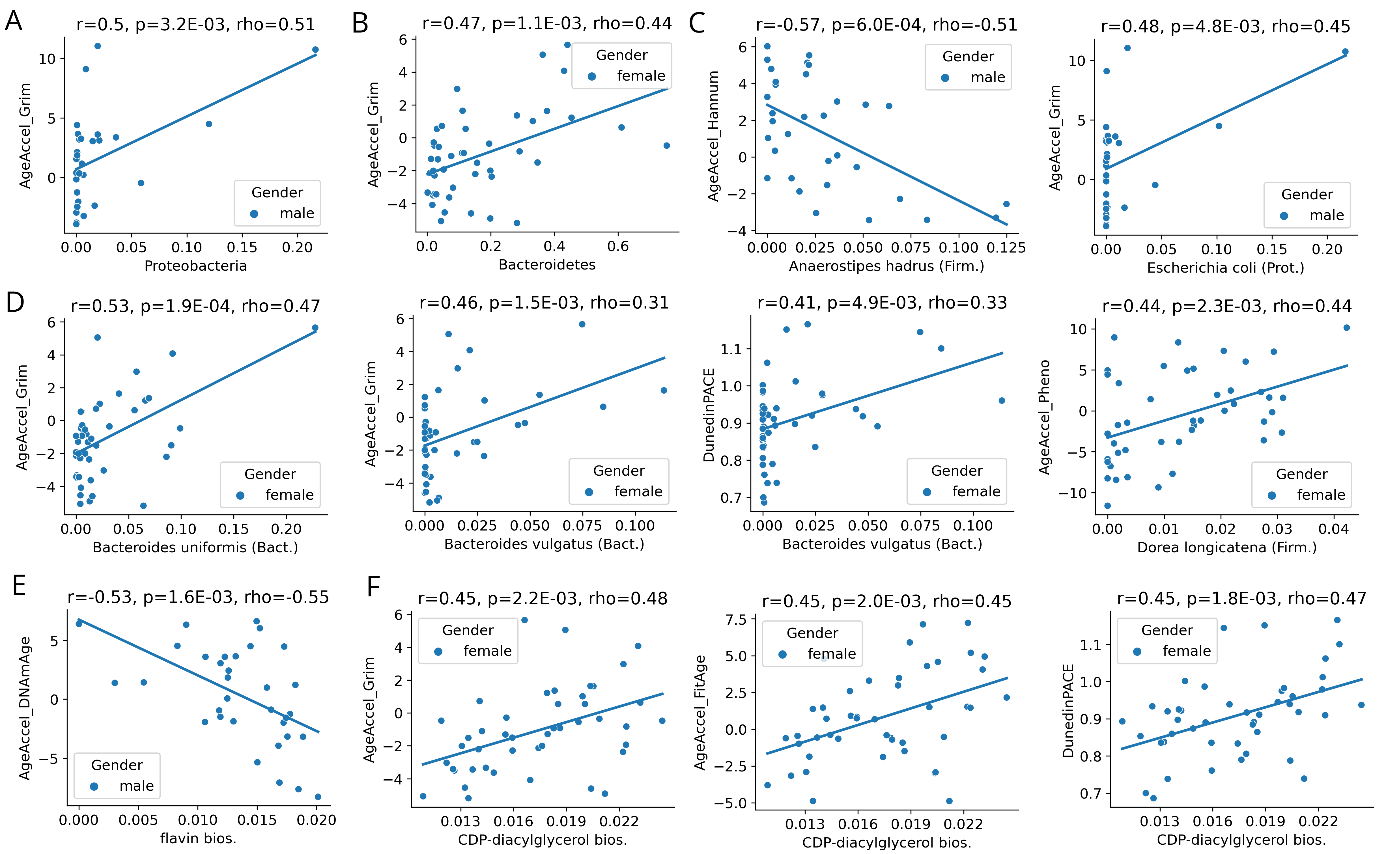
**

**SUPPLEMENTARY FIGURE S3** Strongest associations (**, p <= 0.01) between the gut microbiome and epigenetic clocks (the figure is related to Fig. 2). (A) Phylum level for males. (B) Phylum level for females. (C) Species-level for males. (D) Species-level for females. (E) Pathway analysis for males. (F) Pathway analysis for females. Pearson correlation coefficients (r) the correspondent p-values (p), as well as, Spearman correlation coefficients (rho) are indicated. The regression line (solid blue line) is also shown. Abbreviations are the same as for Fig 2.

**
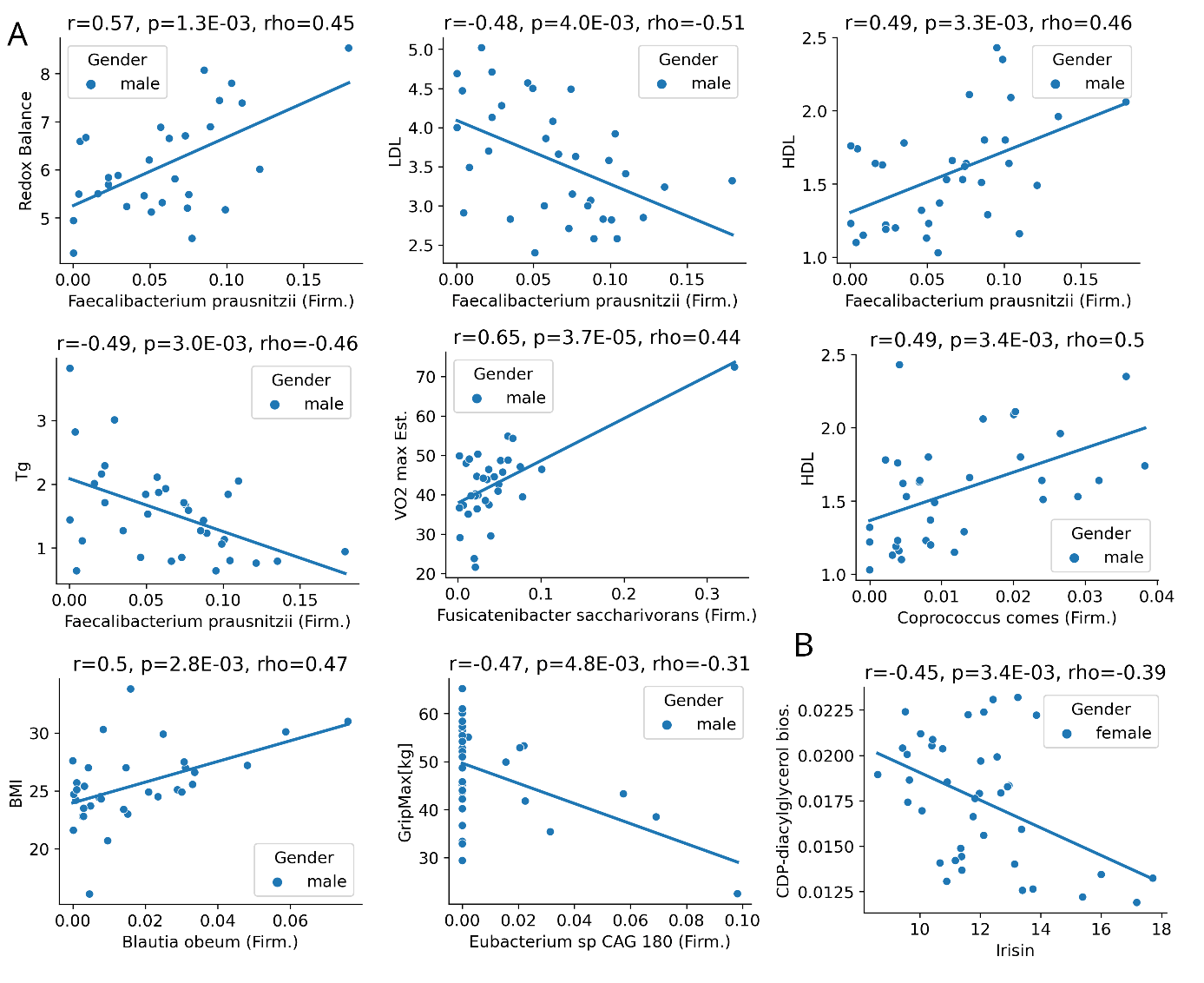
**

**SUPPLEMENTARY FIGURE S4** Strongest associations (**, p < 0.01) between the gut microbiome and exercise-related parameters (associated to Fig. 3). (A) Species-level for males. (B) Pathway analysis for females. Pearson correlation coefficient (r), the correspondent p-value (p) as well as Spearman correlation coefficient (rho) are indicated. The regression line (solid blue line) is also shown. Abbreviations are the same as in Fig 3.

**
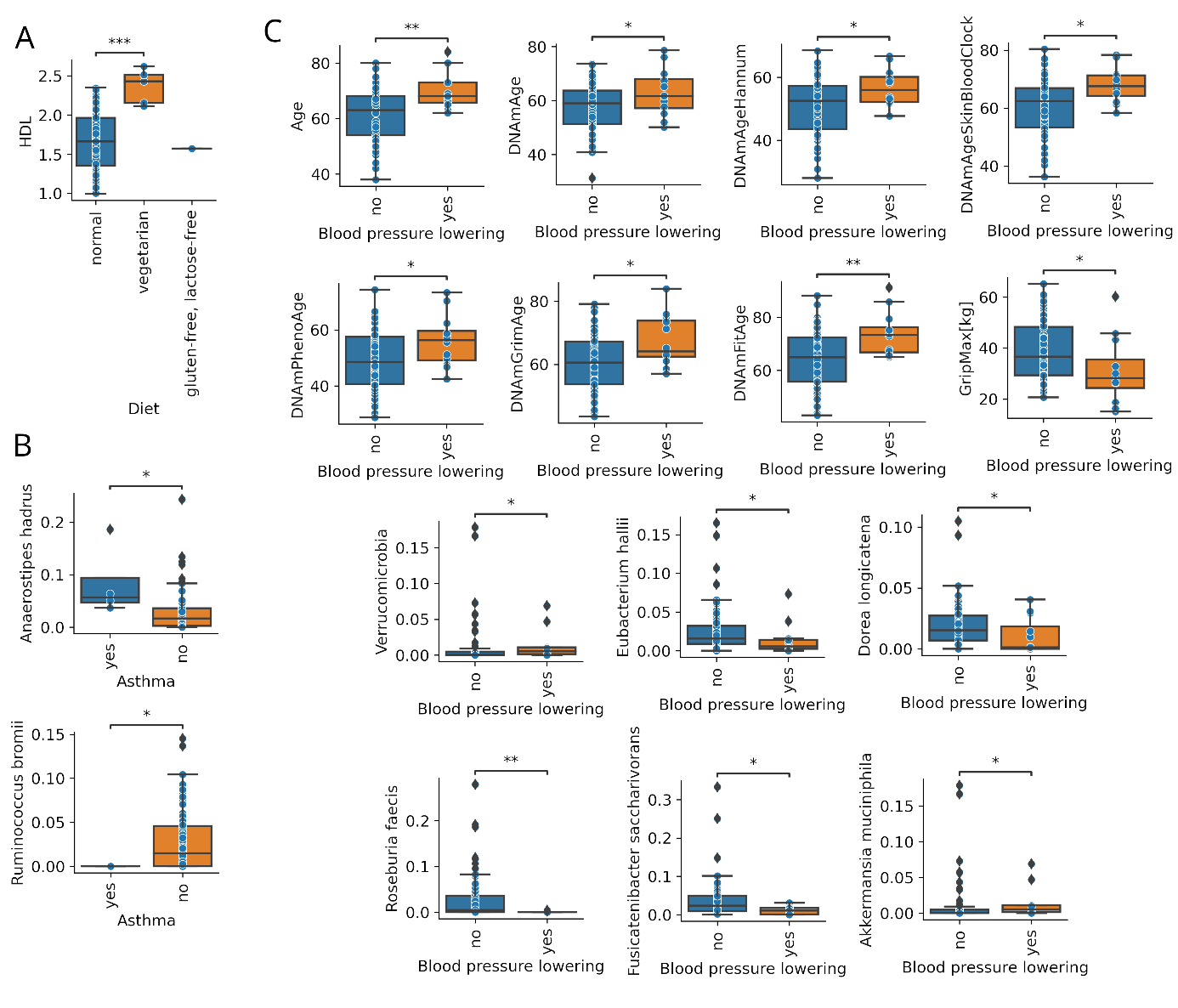
**

**SUPPLEMENTARY FIGURE S5** Associations of exercise-related parameters, aging-clocks (age, age predictions, epigenetic age-accelerations) and the microbiome (species and phylum levels) with (A) diet (B) reported asthma and (C) reported use of a blood pressure lowering medication. Only significant associations are presented**.**
